# Sensors and Sensing for Intelligent Vehicles

**DOI:** 10.3390/s20185115

**Published:** 2020-09-08

**Authors:** David Fernández Llorca, Iván García Daza, Noelia Hernández Parra, Ignacio Parra Alonso

**Affiliations:** Computer Engineering Department, University of Alcalá, 28805 Madrid, Spain; ivan.garciad@uah.es (I.G.D.); noelia.hernandez@uah.es (N.H.P.); ignacio.parrra@uah.es (I.P.A.)

**Keywords:** intelligent vehicles, sensors, sensing, percepction, scene understanding, object detection and tracking, scene segmentation, vehicle positioning, fail-x systems, driver behavior modelling, automatic operation

## Abstract

Over the past decades, both industry and academy have made enormous advancements in the field of intelligent vehicles, and a considerable number of prototypes are now driving our roads, railways, air and sea autonomously. However, there is still a long way to go before a widespread adoption. Among all the scientific and technical problems to be solved by intelligent vehicles, the ability to perceive, interpret, and fully understand the operational environment, as well as to infer future states and potential hazards, represent the most difficult and complex tasks, being probably the main bottlenecks that the scientific community and industry must solve in the coming years to ensure the safe and efficient operation of the vehicles (and, therefore, their future adoption). The great complexity and the almost infinite variety of possible scenarios in which an intelligent vehicle must operate, raise the problem of perception as an "endless" issue that will always be ongoing. As a humble contribution to the advancement of vehicles endowed with intelligence, we organized the Special Issue on Intelligent Vehicles. This work offers a complete analysis of all the mansucripts published, and presents the main conclusions drawn.

## 1. Introduction

When referring to intelligent vehicles, we must be somewhat precise and establish an appropriate definition. The definition of the first word, vehicles, is straightforward. When talking about “vehicles” we can consider different modes of transport, including road vehicles and trains for land transportation, planes for air transportation and ships for water transportation. However, when dealing with the definition and understanding of the second word, intelligence, things get very complicated. Comparing the intelligence of vehicles with the intelligence of humans is one of the most common steps. Still, human intelligence is even more complicated to address and understand than artificial intelligence (e.g., the theory of multiple intelligences). Furthermore, limiting the potential of artificial systems to what humans can do is like driving with only low beam headlights. In fact, although so far intelligent vehicles, in general, have a lower performance than humans (which is reasonable since all the possible components of transportation have been devised for humans), nothing prevents us thinking that in the not-too distant future, they can far exceed the abilities that humans have to drive them.

Intelligent vehicles are highly complex systems designed with a broad set of technological components and sub-systems such as advanced electronics, mechatronics, communications, sensors and actuators, to deal with many different problems including perception, sensing and situation awareness, positioning, localization, navigation and planning, low-level vehicle control, vehicular communications, actuation, trajectory planning, prediction, etc. Among all the problems that intelligent vehicles must solve, the capability to sense, interpret, and fully understand the operational environment, as well as to infer future states and potential hazards, can be considered as the main challenges and, perhaps, the most complex and the most critical tasks to ensure the safety and efficiency of their operation (and, therefore, their future adoption). Perception and scene understanding are probably the main bottlenecks that the scientific community and industry must solve. One of the main questions is what we mean here when we say “solve”, since the variability of possible scenarios in which an intelligent vehicle has to operate is almost infinite. This has been considered as the “never-ending problem”, and it is very reasonable to imagine a future in which advances and improvements in perception systems are always in progress.

**Figure 1:**
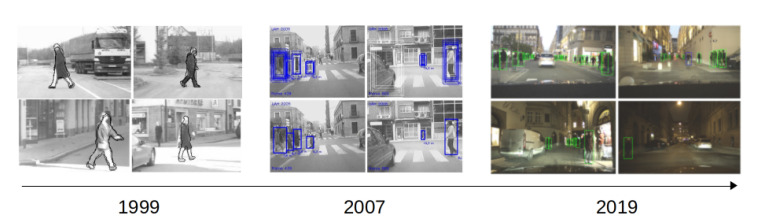
Evolution of pedestrian detection for intelligent vehicles with three examples from [1,2,3].

**Figure 2:**
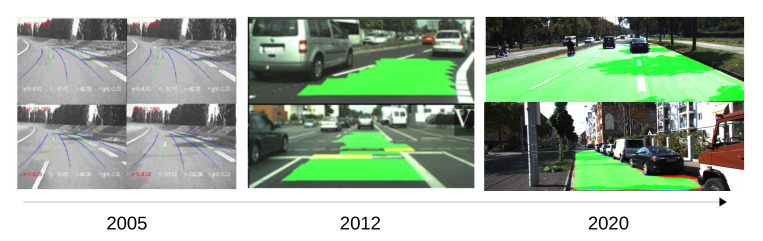
Evolution of road/lane segmentation for intelligent vehicles with three examples from [4,5,6].

Still, during the last decades, both industry and academy have made tremendous advancements in this field, and a considerable number of prototypes are now driving our roads, railways, air and sea autonomously. As an example of the advances, we depict Figure 1 and Figure 2, where we can observe the evolution of approaches such as pedestrian detection and road segmentation.

The essential improvements in sensing for intelligent vehicles and intelligent transportation systems have come from the introduction of deep learning methodologies [7]. However, there is also a current trend focused on reducing the complexity of the models, as well as the dependence on data and learning-based approaches, by combining classical detection methods [8] or using prior information from different sources such as accurate digital maps [9].

In any case, as of today (mid-2020) we can say that intelligent vehicles are not yet above human driving and there is still a long way to go. In these difficult times, it is even more pressing that the transport of people and goods is done in the most automated way possible, allowing social distance when necessary [10], including assistive intelligent transportation systems [11] or even taking into account disruptive solutions [12].

As a humble contribution towards the advancement of vehicles endowed with intelligence, we organized the Special Issue on Intelligent Vehicles. In the next sections we provide a generic description of the Special Issue as well as a brief introduction to each of the manuscripts published in it.

## 2. Special Issue on Intelligent Vehicles

The call for papers of the Special Issue on Intelligent Vehicles was released on February 2019. The main goal of the issue was defined as “to contribute to the state-of-the-art, and to introduce current developments concerning the perception and sensor technologies for intelligent vehicles”. Submissions were accepted until May 2020, a total of 32 manuscripts were finally accepted. The published papers are a well-representative set of current research and developments related to perception and sensor technologies for intelligent vehicles. They include topics such as advance driver assistance systems, automatic vehicle operation, vehicle positioning and localization, fault diagnosis, fail-aware and fail-operational positioning systems, object detection, tracking and prediction, road segmentation, lane detection, traffic lights recognition, smart regenerative braking systems for electric vehicles, driver behavior modeling, simulation-based approaches and intelligent sensing, among others.

**Figure 3:**
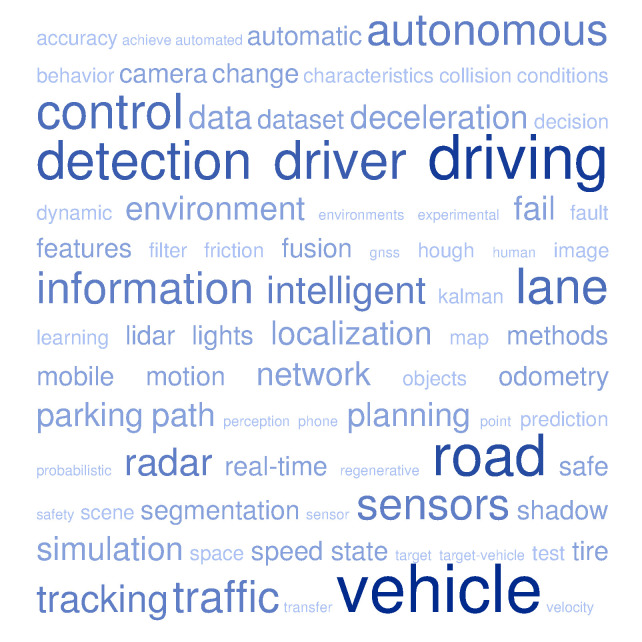
Word cloud from the titles and abstracts of the 32 publications included in the Special Issue (elaborated using tagcrowd).

As an example of the main concepts included in the Special Issue, a word cloud has been elaborated by using the titles and abstracts of the 32 manuscripts, which can be seen in Figure 3.

**Figure 4:**
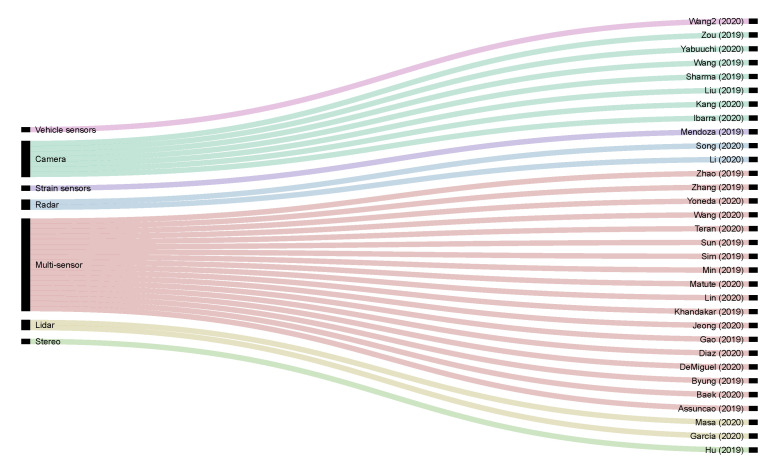
Alluvial diagram showing the correlation between the sensors and the manuscripts.

To provide a better view of the distribution of sensors used in all the papers published in the Special Issue, we present in Figure 4 an alluvial diagram. We can see the correlation between the different proposals and the primary sensor used for each application. Note that, in most cases, the works adopt a multi-sensor approach, and the list of sensors used includes cameras (monocular and stereo), radar, LiDAR, GPS, IMU, vehicle sensors (using OBD interface to read from the CAN Bus), strain sensors, and smartphones.

Concerning the different topics and subtopics, we have identified up to seven main categories, and some sub-categories that are presented in the following list (the number of papers per each category/sub-category is enclosed in parentheses):

- Object detection and scene understanding (11)
  - Vehicle detection and tracking (4): [13,14,15,16].
  - Scene segmentation and interpretation (7)
    * Road segmentation (2): [17,18].
    * Shadow detection (1): [19].
    * Lane detection (2): [20,21].
    * Traffic lights detection (2): [22,23].
- Driver behavior modeling (5)
  - Lane change modeling (2): [24,25].
  - Driver behavior understanding (2): [26,27].
  - Driving behavior for simulation (1): [28].
- Fail-x systems and fault diagnosis (3)
  - Fail-x vehicle positioning (2): [29,30].
  - Fault diagnosis of vehicle motion sensors (1): [31].
- Vehicle positioning and path planning (5)
  - Simultaneous Localization and Mapping (1): [32].
  - Vehicle localization (3): [33,34,35].
  - Path planning (1): [36]
- Smart regenerative braking systems for electric vehicles (2): [37,38].
- Physical intelligence in sensors and sensing (3): [39,40,41]
- Driver assistance systems and automatic vehicle operation (3)
  - Advanced driver assistance systems (1): [42].
  - Automatic parking of road vehicles (1): [43].
  - Automatic train operation (1): [44].

As can be observed, most of the proposals are focused on object detection, scene understanding and vehicle localization, including fail-aware and fail-operational approaches. In the following sections, we provide a detailed description of the papers published in the Special Issue, following the aforementioned categorization.

## 3. Object Detection and Scene Understanding

### 3.1. Vehicle Detection and Tracking

**Figure 5:**
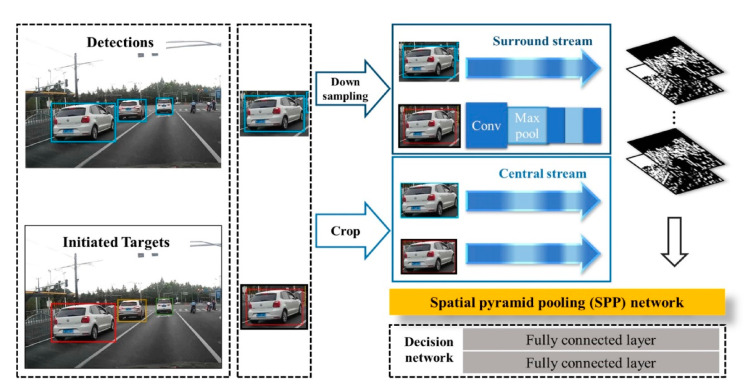
Central-surround two-channel spatial pyramid pooling network (CSTCSPP) based on the Siamese-type architecture (image obtained from [13]).

In [13] a monocular-based real-time multiple vehicles tracking system is proposed by using a novel Siamese network with a spatial pyramid pooling (SPP) layer which is applied to calculate pairwise appearance similarity (see Figure 5). The motion model captured from the bounding boxes provides the relative movements of the vehicles. An online-learned policy treats each tracking period as a Markov Decision Process (MDP) to maintain long-term, robust tracking. The approach achieves significant performance in terms of the “Mostly-tracked”, “Fragmentation”, and “ID switch” variables on the well-known KITTI dataset.

**Figure 6:**
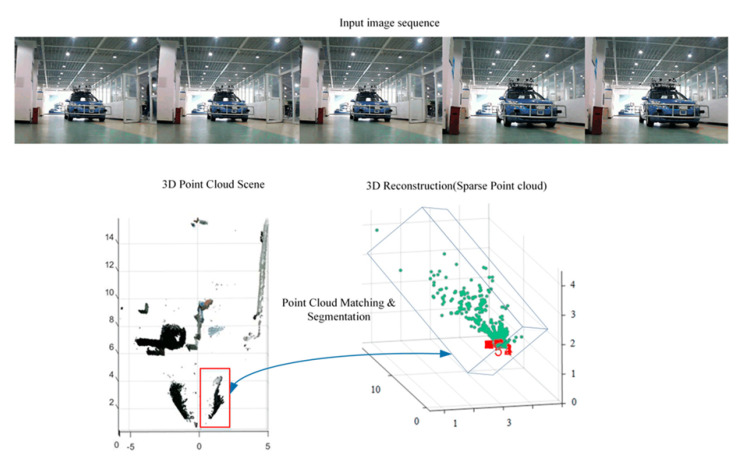
Point cloud matching and segmentation (image obtained from [14]).

Hu et al. propose in [14] an improved edge-oriented segmentation-based method to detect the objects from a 3D point cloud by applying three main steps. First, 2D bounding boxes are selected by edge detection and stixel estimation in 2D images from the stereo system. Second, 3D sparse point clouds are computed in the selected areas. Finally, the dense 3D point clouds of objects are segmented by matching the 3D sparse point clouds of objects with the whole scene point cloud, as can be observed in Figure 6. After comparison with existing segmentation methods, it is demonstrated that the proposed approach improves precision.

**Figure 7:**
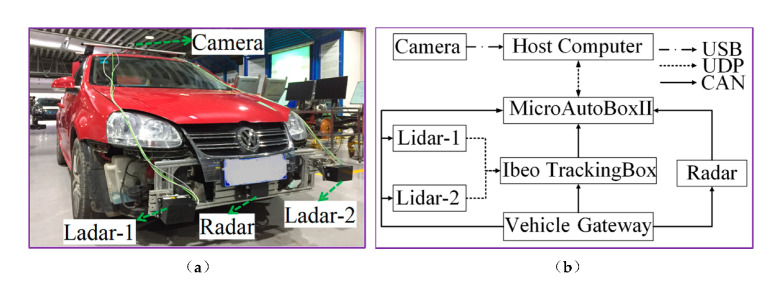
Experimental vehicle. (**a**) Test platform equipment. (**b**) Experimental platform communication (images obtained from [15]).

Song and Wu present in [15] a method to estimate and identify the motion of target-vehicles (surrounding vehicles) using data from a millimeter-wave radar. Based on the Square-Root Cubature Kalman Filter (SRCKF), the Sage–Husa noise statistic estimator (SH-EKF) and the fading memory exponential weighting method are combined to derive a time-varying noise statistic estimator for non-linear systems. This approach is named the Improved Square-Root Cubature Kalman Filter (ISRCKF), and it improves the filtering accuracy of the longitudinal distance and speed about 50% and 25% respectively, with respect to SH-EKF and SRCKF methods. As depicted in Figure 7 the experimental platform used during the experiments includes one front radar to get raw data, one camera to obtain images from the scenes, and two LIDAR sensors to generate the ground truth. The classification and recognition results of the target-vehicle motion state are consistent with the actual target-vehicle motion state.

**Figure 8:**
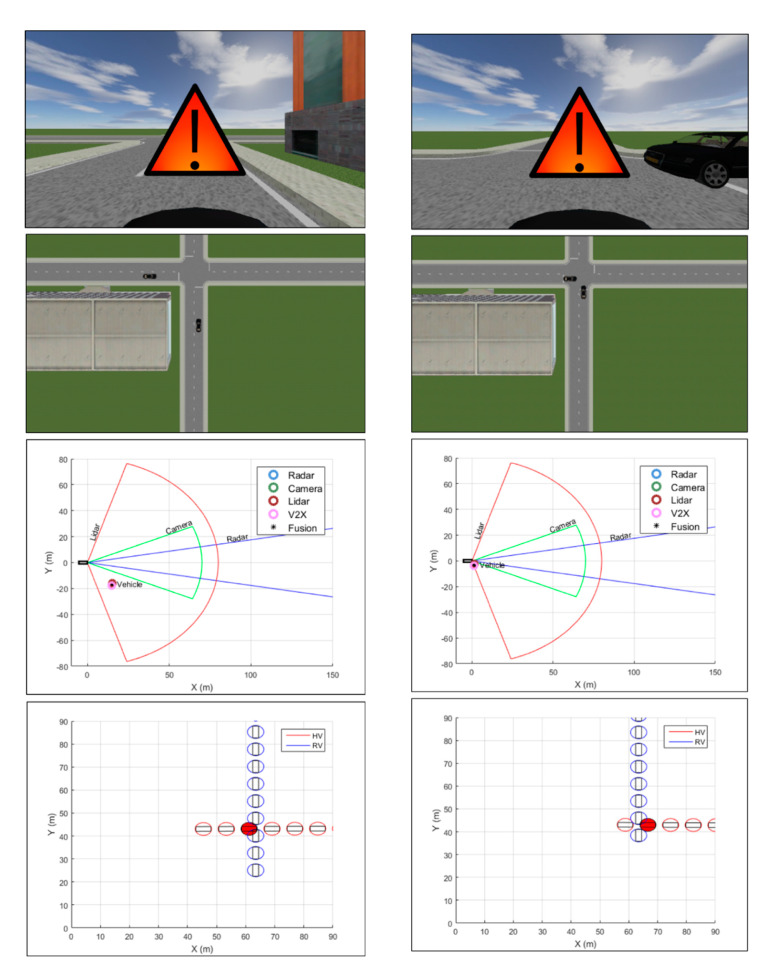
Vehicle–vehicle collision simulation results and snapshots of the experimental environment at two different time points (images obtained from [16]).

In [16] a vehicle collision warning system is proposed based on a Kalman filter-based approach for high-level fusion of multiple sensors, including radar, LIDAR, camera and wireless communication. The trajectories of remote targets are predicted, and an appropriate warning to the driver is provided based on the TTC (Time-To-Collision) estimate and the risk assessment. The multi-sensor approach is validated using a virtual driving simulator (see Figure 8) with two different Euro NCAP test scenarios: a vehicle–vehicle and a vehicle–pedestrian collision scenarios.

### 3.2. Scene Segmentation and Interpretation

**Figure 9:**
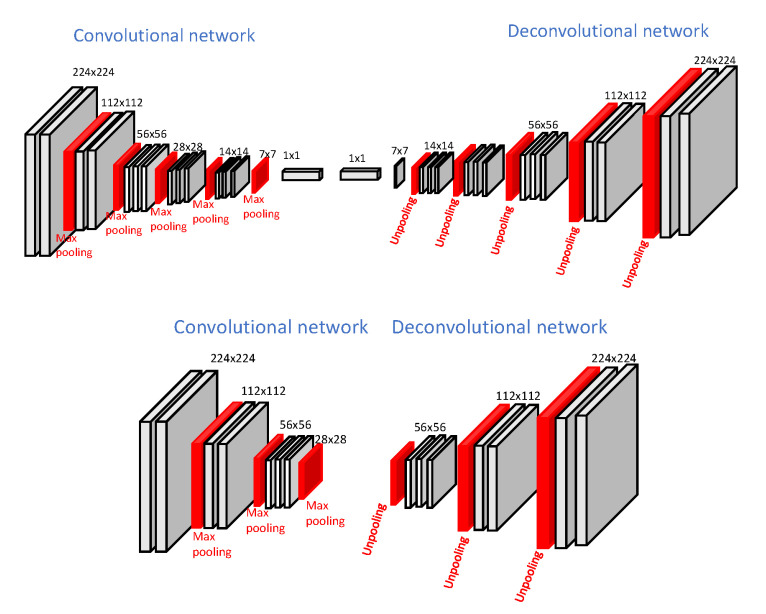
**Top**: Original DeconvNet architecture, **Bottom**: Proposed light-weight network architecture (images obtained from [17]).

The availability of large training dataset demanded by deep learning algorithms is not always possible. One potential solution is transfer learning from different domains. However, this method may not be a straightforward task considering issues such as original network size or large differences between the source and target domains. In [17], transfer learning is applied for semantic segmentation of off-road driving environments using a lightweight deconvolutional network (depicted in Figure 9) which is half the size of the original DeconvNet architecture. Transfer learning and fine-tuning is applied from the original pre-trained DeconvNet to the lightweight version. In addition, a synthetic dataset is used as an intermediate domain. It is observed that fine-tuning the model trained with the synthetic dataset that simulates the off-road driving environment provides more accurate results for the segmentation of real-world off-road driving environments than transfer learning without using a synthetic dataset does, as long as the synthetic dataset is generated considering real-world variations.

**Figure 10:**
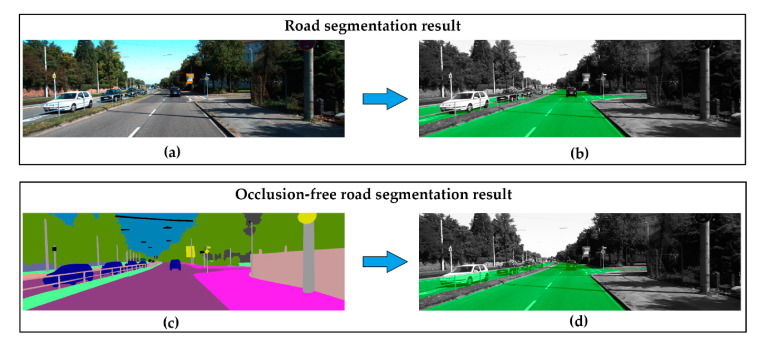
Comparison of road segmentation and proposed occlusion-free road segmentation. (**a**) RGB image; (**b**) standard road segmentation; (**c**) semantic segmentation; (**d**) occlusion-free road segmentation (images obtained from [18]).

Wang et al. address in [18] the problem of performing semantic segmentation for occluded areas (the overall idea is depicted in Figure 10). This is a complex problem that requires a comprehensive understanding of the geometry and the semantics of the visible environment. A specific dataset (KITTI-occlusion-free road segmentation) based on KITTI dataset is created and a specific lightweight fully convolutional neural network, named OFRSNet (occlusion-free road segmentation network), is designed to predict occluded portions of the road in the semantic domain by looking around foreground objects and visible road layout. The global context module is used to build up the down-sampling and joint context up-sampling block in the network and a spatially-weighted cross-entropy loss is used. Extensive experiments on different datasets verify the effectiveness of the proposed methodology, and comparisons with current methods show that the proposed approach outperforms the baseline models by obtaining a better trade-off between accuracy and computational cost, which makes the presented approach appropriate to be applied to intelligent vehicles in real-time.

**Figure 11:**
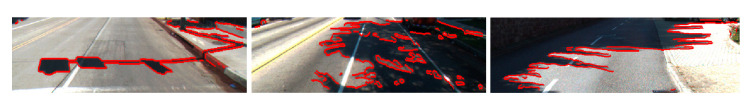
Examples of the results of the shadow edge detection method (images obtained from [19]).

One of the most challenging vision-based perception problems is to mitigate the effects of shadows on the road, which increases the difficulty of fundamental tasks such as road segmentation or lane detection. In [19], a new shadow detection method is proposed based on the skylight and sunlight contributions to the road surface chromaticity. Six constraints on shadow and non-shadowed regions are derived from these properties. The chrominance properties and the associated constraints are used as shadow features in an effective shadow detection method intended to be integrated on an onboard road detection system where the identification of cast shadows on the road is a determinant stage. The underlying principle is based on the following assumption: a non-shadowed road region is illuminated by both skylight and sunlight, whereas a shadowed region is illuminated by skylight only and, thus, their chrominance values vary. Shadow edges are detected and classifying by verifying whether the pixel chrominance values of regions on both sides of the edges satisfy up to six different constraints. Experiments on real traffic scenes (some examples can be seen in Figure 11) demonstrated the effectiveness of the proposed shadow detection method, outperforming previous approaches based on physical features.

**Figure 12:**
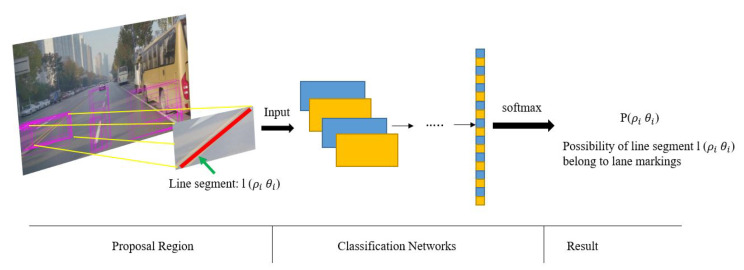
Line segments classified by the proposed network into the probabilistic Hough space which records the the confidence probability of each line segment (image obtained from [20]).

In [20] a novel approach based on multiple frames is proposed by taking advantage of the fusion of vision and Inertial Measurement Units (IMU). Hough space is employed as a storage medium where lane markings can be stored and visited conveniently. A CNN-based classifier is introduced to measure the confidence probability of each line segment, and transforms the basic Hough space into a probabilistic Hough space, as depicted in Figure 12. Pose information provided by the IMU is applied to align previous probabilistic Hough spaces to the current one and a filtered probabilistic Hough space is acquired by smoothing the primary probabilistic Hough space across frames. The proposed approach is applied experimentally, and the results demonstrate a satisfying performance compared to various existing methods.

**Figure 13:**
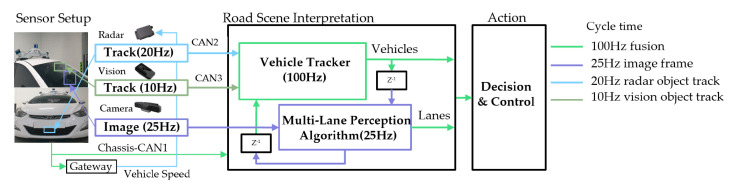
Multi-sensor setup and fusion chart (image obtained from [21]).

Still, lane detection and tracking in a complex road environment is one of the most important research areas in highly automated driving systems. Studies on lane detection cover a variety of difficulties, such as shadowy situations, dimmed lane painting, and obstacles that prohibit lane feature detection. Jeong et al. have carefully selected typical scenarios in which the extraction of lane candidate features can be easily corrupted by road vehicles and road markers that lead to degradation in the road scene understanding [21]. They introduced a novel framework combining a lane tracker method integrated with a camera and a radar forward vehicle tracker system, which is especially useful in dense traffic situations. An image template occupancy matching method is integrated with the vehicle tracker which allows to avoid extracting irrelevant lane features caused by forward target vehicles and road markers. In addition, a robust multi-lane detection method is presented by tracking adjacent and ego lanes. The proposed approach is comprehensively evaluated using a real dataset comprised of difficult and complex road scenarios recorded with a experimental vehicles with an appropriate sensor setup (see Figure 13). Experimental result shows that the proposed method is reliable for multi-lane detection at the presented difficult situations.

**Figure 14:**
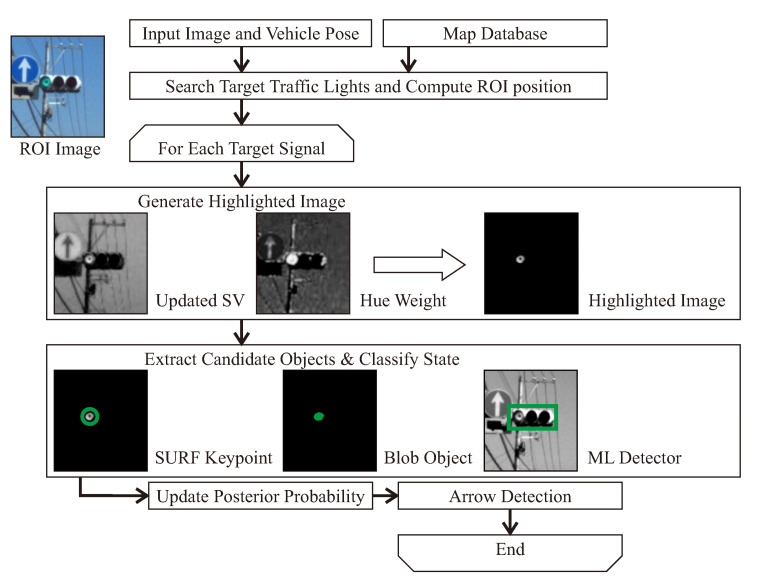
Flowchart of the method proposed in [22].

Another important type of object that should be identified by intelligent vehicles in urban areas are the traffic lights. In [22] traffic lights and arrow lights are recognized by image processing using digital maps and precise vehicle positioning. The overall approach is shown in Figure 14. The use of a digital map allows the determination of a region-of-interest (ROI) in the image to reduce the computational cost and false alarms. In addition, this study develops an algorithm to recognize arrow lights using relative positions of traffic lights which allows for the recognition of far distant arrow lights that are difficult for humans to see clearly. Quantitative evaluations indicate that the proposed method achieved 91.8% and 56.7% of the average f-value for traffic lights and arrow lights, respectively. The proposed arrow-light detection method can recognize small arrow objects even for sizes smaller than 10 pixels. The experiments indicate that the performance of the proposed method meets the necessary requirements for smooth acceleration/deceleration at intersections in automated driving.

**Figure 15:**
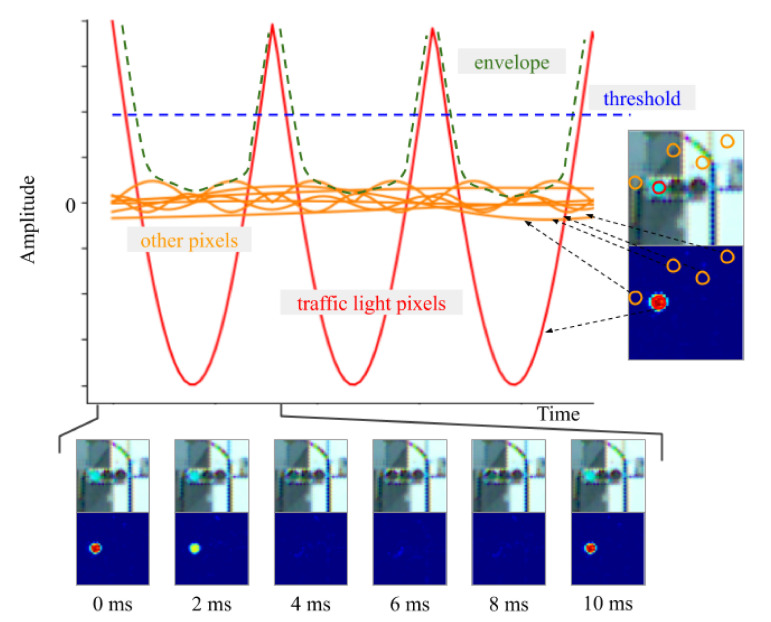
Brightness variation (blinking) for each pixel can be captured by the high-speed camera (images obtained from [23]).

Yabuuchi et al. address the problem of detecting LED traffic lights which blink at high frequencies using a high-speed camera [23]. The method is composed of six modules, which includes a band-pass filter and a Kalman filter. All modules run simultaneously to achieve real-time processing. Actually, they can run up to 500 FPS for images with a resolution of 800 × 600 pixels. The proposed technique is robust under various illuminations because it can detect traffic lights by extracting information from the blinking pixels at a specific frequency (see Figure 15). An original dataset was created with the high-speed camera including images under different illumination conditions such as a sunset or night scene. The results show that the system can detect traffic lights with a different appearance. The most important benefits of this work are that neither parameter-tuning nor learning from a dataset are needed.

## 4. Driver Behavior Modeling

Studying and modeling drivers behaviors is a common practice to address motion and action recognition and prediction of surrounding vehicles, as well as to deal with advanced driver assistance systems in the ego vehicle. One of the most risky maneuvers of road vehicles is lane change. Most lane-changing models deal with lane-changing maneuvers solely from the merging driver’s standpoint and thus ignore driver interaction. To overcome this shortcoming, in [24] a game-theoretical decision-making model is developed. Validation makes use of empirical merging maneuver data at a freeway on-ramp. Specifically, this paper advances the repeated game model by using updated payoff functions. Validation results using the Next Generation SIMulation (NGSIM) empirical data show that the developed game-theoretical model provides better prediction accuracy compared to previous work, giving correct predictions approximately 86% of the time. The proposed lane change model, which captures the collective decision-making between human drivers, can be applied to develop automated vehicle driving strategies.

**Figure 16:**
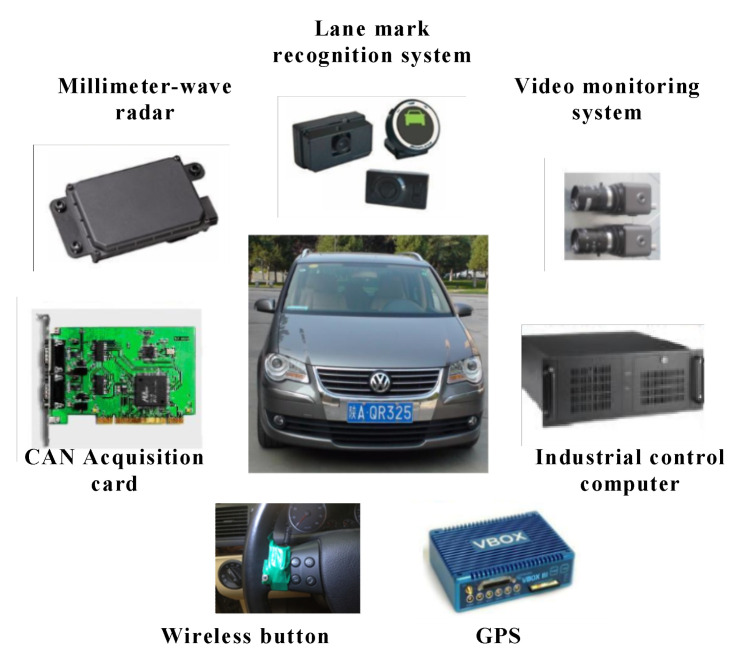
Experimental vehicle used to validate the proposed lane-change decision model in [25].

Determining an appropriate time to execute a lane change is a critical issue for the development of autonomous vehicles. However, few studies have considered the rear and the front vehicle-driver’s risk perception while developing a human-like lane-change decision model. In [25], Wang et al. propose a lane-change decision model by identifying a two level threshold that conforms to a driver’s perception of the ability to safely change lanes with a rear vehicle approaching fast. Based on the signal detection theory and extreme moment trials on a real highway, two thresholds of safe lane change are determined with consideration of risk perception of the rear and the subject vehicle drivers, respectively. The rear vehicle’s Minimum Safe Deceleration (MSD) during the lane change maneuver of the subject vehicle is selected as the lane change safety indicator, and it is calculated using the human-like lane-change decision model. The results of this paper show that, compared with the driver in the front extreme moment trial, the driver in the rear extreme moment trial is more conservative during the lane change process. To meet the safety expectations of the subject and rear vehicle drivers, the primary and secondary safe thresholds were determined to be 0.85 m/s${}^{2}$ and 1.76 m/s${}^{2}$, respectively. A multi-sensor platform was used on an experimental vehicle to record the data and validate the system (see Figure 16). The proposed decision model can be of great help to make intelligent vehicles safer and more polite during lane changes, improving acceptance and safety.

**Figure 17:**
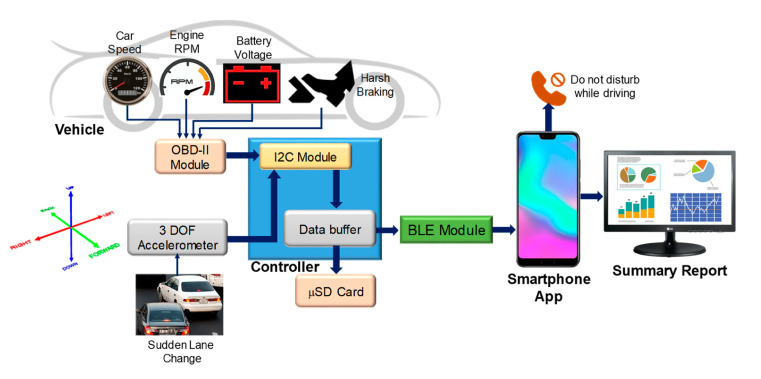
Complete block system diagram proposed in [26].

As mentioned above, understanding the driver’s behavior is a key factor for assistance systems, but also for monitoring the state of the driver for automated driving with SAE levels 2 or 3. The use of the phone is of particular interest to prevent drivers from being distracted. As stated in [26], recent studies have shown that 70% of the young and aware drivers are used to texting while driving. There are many different technologies used to control mobile phones while driving, including electronic device control, global positioning system (GPS), on-board diagnostics (OBD)-II-based devices, etc. However, we can even think of mobile phones as a sensor device to be used inside the vehicle to monitor the driver’s behavior. These devices acquire vehicle information such as the car speed and use the information to control the driver’s phone such as preventing them from making or receiving calls at specific speed limits. The information from the devices is interfaced via Bluetooth and can later be used to control mobile phone applications. The main aim of the work presented in [26] is to design a portable system (the overall structure is depicted in Figure 17) for monitoring the use of a mobile phone while driving and for controlling the driver’s mobile phone, if necessary, when the vehicle reaches a specific speed limit (>10 km/h). A paper-based self-reported questionnaire survey was carried out among 600 teenage drivers from different nationalities to see the driving behavior of young drivers in Qatar. A mobile application is presented to monitor the mobile usage of a driver and a OBD-II module-based portable system was designed to acquire data from the vehicle to identify drivers’ behavior with respect to phone usage, sudden lane changes, and abrupt breaking/sharp speeding. The presented application, which combines the sensors of the mobile phone and the vehicle, can significantly improve drivers’ behavior.

Assuncao et al. propose in [27] the use of the Statistical Process Control (SPC) and Exponentially Weighted Moving Average methods for the monitoring of drivers using approaches based on the vehicle and the driver’s behavior. Different detection methods are independently devised for lane departure, sudden driver behaviors and driver fatigue. All of them consider information from sensors scattered by the vehicle in a multi-sensor fashion. The results show the efficiency of the proposed approach. Lane departure detection obtained results of up to 76.92% (without constant speed) and 84.16% (speed maintained at 60 kmh aprox.). Furthermore, sudden movements detection obtained results of up to 91.66% (steering wheel) and 94.44% (brake). The driver fatigue is detected in up to 94.46% situations.

**Figure 18:**
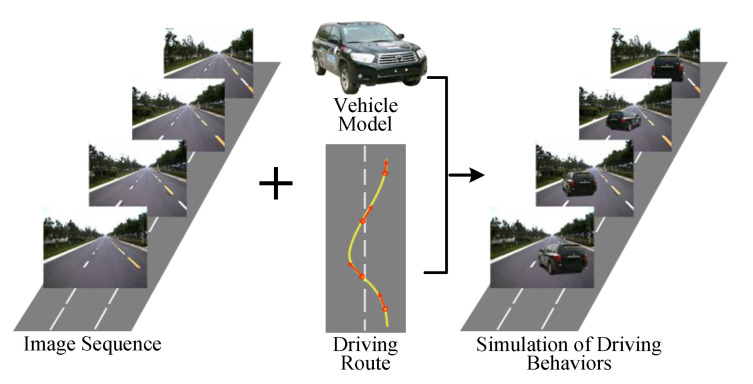
Simulation of driving behaviors with image sequences collected from real road environment (image obtained from [28]).

The use of simulation is also getting more attention in the research community and industry. In [28] an automatic approach of simulating dynamic driving behaviors of vehicles in traffic scene represented by image sequences is proposed (see Figure 18). The spatial topological attributes and appearance attributes of virtual vehicles are computed separately, according to the constraint of geometric consistency of sparse 3D space organized by image sequence. To achieve this goal, three main problems must be solved. First, registration of vehicle in a 3D space of road environment. Second, to generate the vehicle’s image observed from corresponding viewpoint in the road scene. Third, to maintain consistency between the the vehicle and the road environment. After the proposed method was embedded in a scene browser, a typical traffic scene including the intersections is chosen for a virtual vehicle to execute the driving tasks of lane change, overtaking, slowing down and stop, right turn, and U-turn. The experimental results show that different driving behaviors of vehicles in typical traffic scenes can be exhibited smoothly and realistically. The proposed method can also be used for generating simulation data of traffic scenes that are difficult to collect in real driving scenarios.

## 5. Fail-X Systems and Fault Diagnosis

Robust sensing under different lighting and weather conditions, and considering all the complexity that the vehicle can face in real world scenarios becomes mandatory. To that end, it is fundamental to advance towards fail-aware, fail-safe, and fail operational systems, including fault diagnosis.

**Figure 19:**
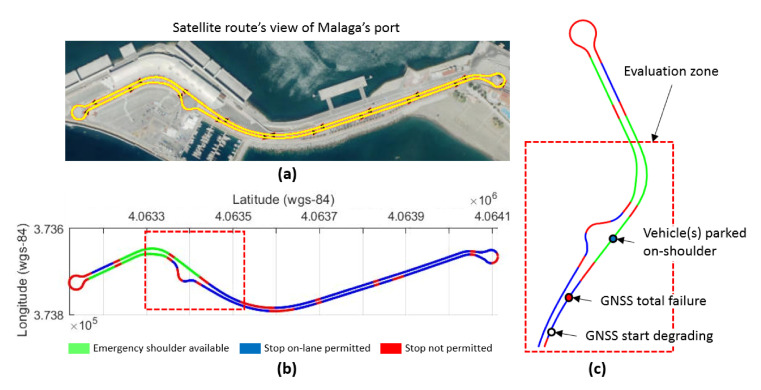
Realistic environment scenario for automated driving system tests on simulation. (**a**) Satellite’s view of urban route, (**b**) permitted and non-permitted stops in case of total positioning failure, and (**c**) evaluation zone for test case study. (image obtained from [29]).

As explained in [29], presently, in the event of a failure in automated driving systems, control architectures rely on hardware redundancies over software solutions to assure reliability, or wait for human interaction in takeover requests to achieve a minimal risk condition. As user confidence and final acceptance of autonomous vehicles are strongly related to safety, automated fall-back strategies must be assured as a response to failures while the system is performing a dynamic driving task. In the work presented by Matute-Peaspan et al. [29], a fail-operational control architecture approach and dead-reckoning strategy in case of positioning failures are developed. A fail-operational system is capable of detecting failures in the last available positioning source, warning the decision stage to set up a fall-back strategy and planning a new trajectory in real time. The surrounding objects and road borders are considered during the vehicle motion control after failure, to avoid collisions and lane-keeping purposes. A case study based on a realistic urban scenario (depicted in Figure 19) is simulated for testing and system verification, showing that the proposed approach always bears in mind both the passenger’s safety and comfort during the fall-back maneuvering execution.

**Figure 20:**
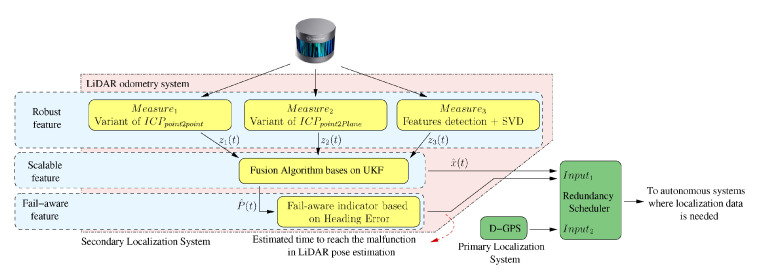
General diagram. The developed blocks are represented in yellow. The horizontal blue strips represent the main features of the odometry system. A framework where the LiDAR odometry system can be integrated within the autonomous driving cars topic is depicted with green blocks, such as a secondary localisation system (image obtained from [30]).

García-Daza et al. state in [30] that, currently, even the most advanced architectures require driver intervention when functional system failures or critical sensor operations take place, presenting problems related to driver state, distractions, fatigue, and other factors that prevent safe control. All odometry systems have drift error, making it difficult to use them for localization tasks over extended periods. In this work, a redundant, accurate and robust LiDAR odometry system with specific fail-aware features that can allow other systems to perform a safe stop man oeuvre without driver mediation is presented. A fail-aware indicator is designed which estimates a time window in which the system can manage the localization tasks appropriately. The odometry error is minimized by applying a dynamic 6-DoF model and fusing measures based on the Iterative Closest Points (ICP), environment feature extraction, and Singular Value Decomposition (SVD) methods. A general overview of the proposed system can be seen in Figure 20. The obtained results are promising for two reasons. First, in the KITTI odometry data set, the ranking achieved by the proposed method is twelfth, considering only LiDAR-based methods, where its translation and rotation errors are 1% and 0.0041 deg/m, respectively. Secondly, the encouraging results of the fail-aware indicator demonstrate the safety of the proposed LiDAR odometry system. The results depict that, in order to achieve an accurate odometry system, complex models and measurement fusion techniques must be used to improve its behavior. Furthermore, if an odometry system is to be used for redundant localization features, it must integrate a fail-aware indicator for use in a safe manner.

Sensor fault diagnosis is a necessary step to design fail-x systems. In [31] a fault diagnosis logic and signal restoration algorithms for vehicle motion sensors are presented. The primary idea of the proposed fault detection system is the conversion of measured wheel speeds into vehicle central axis information and the selection of a reference central axis speed based on this information. Thus, the obtained results can be employed to estimate the speed for all wheel sides, which are compared with measured values to identify faults and recover the fault signal. For fault diagnosis logic, a conditional expression is derived with only two variables to distinguish between normal and fault. Further, an analytical redundancy structure and a simple diagnostic logic structure are proposed. Finally, an off-line test is conducted using test vehicle information to validate the proposed method. It demonstrates that the proposed fault detection and signal restoration algorithm can satisfy the control performance required for each sensor failure.

## 6. Vehicle Positioning and Path Planning

One of the key features needed to perform autonomous navigation is to have accurate global locations of the vehicle. Vehicle positioning is a fundamental task for long-term navigation in a digital map, but also to perform short-term path planning in the local environment. In the previous section, we showed two manuscripts related with fail-x vehicle positioning [29,30]. In this section we present five more papers that deal with this important topic.

In [32], an enhanced visual SLAM algorithm based on the sparse direct method is proposed to deal with illumination sensitivity problems of mobile ground equipment. The presented procedure can be described as follows. First, the vignette and response functions of the input sequences are optimized based on the photometric formation of the camera. Second, the Shi–Tomasi corners of the input sequence are tracked, and optimization equations are established using the pixel tracking of sparse direct visual odometry (VO). Third, the Levenberg–Marquardt (L–M) method is applied to solve the joint optimization equation, and the photometric calibration parameters in the VO are updated to realize the real-time dynamic compensation of the exposure of the input sequences, thus reducing the effects of the light variations on accuracy and robustness. Finally, a Shi–Tomasi corner filtered strategy is designed to reduce the computational complexity of the proposed algorithm, and the loop closure detection is realized based on the oriented FAST and rotated BRIEF (ORB) features. The proposed algorithm is tested using TUM, KITTI, EuRoC, and a specific environment. Experimental results show that the positioning and mapping performance of the proposed algorithm is promising.

Lin et al. propose in [33] a sensor fusion approach to reduce typical problems of global positioning system (GPS) such as noisy signal and multi-path routing in urban environments. To localize the vehicle position, a particle-aided unscented Kalman filter (PAUKF) algorithm is proposed. The Unscented Kalman Filter (UKF) updates the vehicle state, which includes the vehicle motion model and non-Gaussian noise affection. The Particle Filter (PF) provides additional updated position measurement information based on an onboard sensor and a high definition (HD) digital map. This methodology is validated in a simulated environment. The obtained results show that this method achieves better precision and comparable stability in localization performance compared to previous approaches.

**Figure 21:**
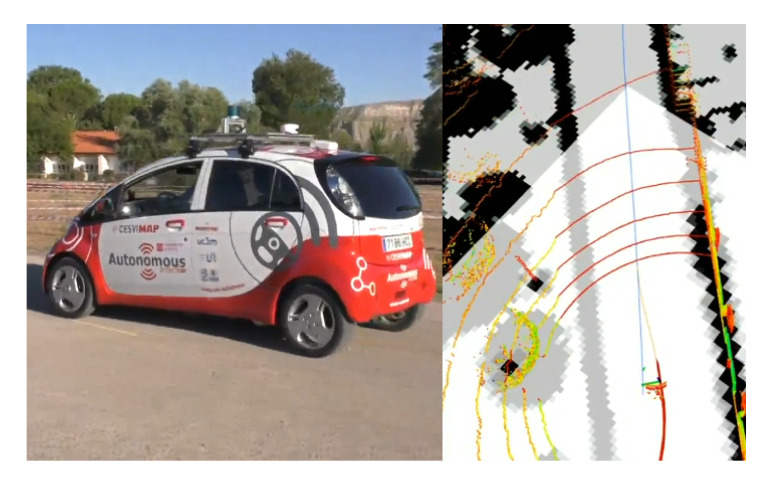
(**Left**) Autonomous vehicle platform used during the experiments. (**Right**) Visualization of the LiDAR point cloud (images obtained from [34]).

In [34], the authors propose a method that improves autonomous vehicle positioning using a modified version of the probabilistic laser localization like the Monte Carlo Localization (MCL) algorithm. The weights of the particles are enhanced by adding Kalman filtered Global Navigation Satellite System (GNSS) information. GNSS data are used to improve localization accuracy in places with fewer map features and to prevent the kidnapped robot problems. Besides, laser information improves accuracy in places where the map has more features and GNSS higher covariance, allowing the approach to be used in specifically difficult scenarios for GNSS such as urban canyons. The algorithm is tested using the KITTI odometry dataset proving that it improves localization compared with classic GNSS + Inertial Navigation System (INS) fusion and Adaptive Monte Carlo Localization (AMCL). The presented approach is also tested in the autonomous vehicle platform of the Intelligent Systems Lab (LSI), of the University Carlos III of Madrid (depicted in Figure 21), providing promising qualitative results.

Diaz-Arango et al. propose in [36] a multiple-target collision-free path planning based on homotopy continuation capable to calculate a collision-free path in a single execution for complex environments. The method exhibits better performance, both in speed and efficiency, and robustness compared to the original Homotopic Path Planning Method (HPPM). Among the new schemes that improve their performance are the Double Spherical Tracking (DST), the dummy obstacle scheme, and a systematic criterion to a selection of repulsion parameter. The case studies, although focusing on robotics indoor environments, show the efficiency to find a solution path in just a few milliseconds, even if they have narrow corridors and hundreds of obstacles. Additionally, a comparison between the proposed method and sampling-based planning algorithms (SBP) with the best performance is presented. The method exhibits better performance than SBP algorithms for execution time, memory, and, in some cases, path length metrics. To validate the feasibility of the computed paths two simulations using the pure-pursuit controlled and differential drive robot model contained in the Robotics System Toolbox of MATLAB are presented. The proposed approach will be further applied and validated in intelligent vehicle environments.

Autonomous racing provides very similar technological issues than standard autonomous driving while allowing for more extreme conditions in a safe human environment. In [35] a localization architecture for a racing car that does not rely on Global Navigation Satellite Systems (GNSS) is presented. More specifically, two multi-rate Extended Kalman Filters and an extension of a state-of-the-art laser-based Monte Carlo localization approach that exploits some prior knowledge of the environment and context are combined. When driving near the friction limits, localization accuracy is critical as small errors can induce large errors in control due to the nonlinearities of the vehicle’s dynamic model. The proposed method is compared with a solution based on a widely employed state-of-the-art implementation, outlining its strengths and limitations within the experimental scenario. The architecture is then tested both in simulation and experimentally on a full-scale autonomous electric racing car during an event of Roborace Season Alpha. The results show its robustness in avoiding the robot kidnapping problem typical of particle filters localization methods, while providing a smooth and high rate pose estimate. The pose error distribution depends on the car velocity, and spans on average from 0.1 m (at 60 km/h) to 1.48 m (at 200 km/h) laterally and from 1.9 m (at 100 km/h) to 4.92 m (at 200 km/h) longitudinally.

## 7. Smart Regenerative Braking Systems

**Figure 22:**
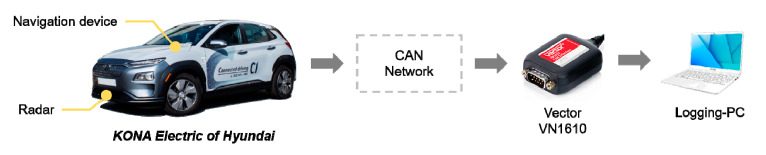
Test vehicle, sensors and logging system used by [37].

Two manuscripts from the same research group have been published in the Special Issue which provides classification and prediction approaches to deal with regenerative braking for Electric Vehicles (EV). Smart regenerative braking systems are an autonomous version of one-pedal driving in EVs. To implement them, a deceleration planning algorithm is necessary to generate the deceleration used in automatic regenerative control. To reduce the discomfort from the automatic regeneration, the deceleration should be similar to human driving. In [37], a deceleration planning algorithm based on Multi-Layer Perceptron (MLP) is proposed. The MLP models can mimic human driving behavior by learning the driving data. In addition, the proposed deceleration planning algorithm has a classified structure to improve the planning performance in each deceleration condition. Therefore, the individual MLP models are designed according to three different deceleration conditions: car-following, speed bump, and intersection. The proposed algorithm is validated through driving simulations using a test vehicle equipped with multiple sensors and logging software (see Figure 22). Time to collision (TTC) and similarity to human driving is analyzed. The results show that the minimum TTC was 1.443 s and the velocity root-mean-square error (RMSE) with human driving was 0.302 m/s.

**Figure 23:**
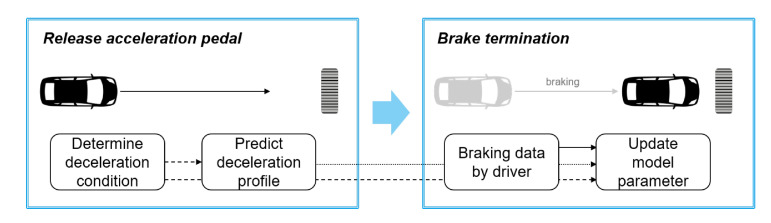
Overview of the methodology presented in [38].

The vehicle state prediction on decelerating driving conditions can be applied to automatic regenerative braking in EVs. However, drivers can feel a sense of heterogeneity when regenerative control is performed based on prediction results from a general prediction model. As a result, a deceleration prediction model which represents individual driving characteristics is required to ensure a more comfortable experience with automatic regenerative braking control. Thus, in [38], a deceleration prediction model based on the parametric mathematical equation and explicit model parameters is presented. The model is designed specifically for deceleration prediction by using the parametric equation that describes deceleration characteristics. Furthermore, the explicit model parameters are updated according to individual driver characteristics using the driver’s braking data during real driving situations. An overview of the proposed methodology is depicted in Figure 23. The method is integrated and validated on a real-time embedded system, and it is applied to the model-based regenerative control algorithm as a case study.

## 8. Physical Intelligence in Sensors and Sensing

**Figure 24:**
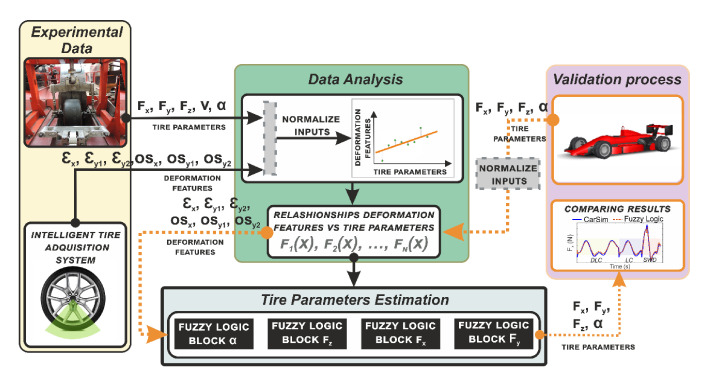
Overview of the methodology presented in [39] to develop the strain-based tire state estimation system.

Adding intelligence to some important components and sensors of the vehicles is a fundamental approach to increase the intelligence of the vehicles, providing new measurements and variables very useful for higher-level decision modules. For example, in [39] the possibility of using tires as active sensors is explored using strain sensors. The concept is known as Intelligent or Smart Tires and can provide relevant vehicle dynamics information. The main goal of this work is to estimate all tire forces, based only on deformations measured in the contact patch. Through an indoor test rig data, an algorithm is developed to select the most relevant features of strain data and correlate them with tire parameters. Tire contact patch information is transmitted to a fuzzy logic system to estimate the tire parameters (see Figure 24 for an overview of the proposed system architecture). To evaluate the reliability of the proposed estimator, the well-known simulation software CarSim is used to back up the estimation results. The obtained estimations are checked with the simulation results enabling the behavior of the intelligent tire to be tested for different maneuvers and velocities. The proposed methodology provides key information about the tire parameters directly from the only contact that exists between the vehicle and the road.

Gao et al. propose in [40] a nonlinear observer aided by vehicle lateral displacement information for estimating the road friction coefficient, which is a key parameter for autonomous vehicles and vehicle dynamic control. A modified version of the tire brush model is proposed to describe the tire characteristics more precisely in high friction conditions using tire test data. Then, on the basis of vehicle dynamics and a kinematic model, a nonlinear observer is designed, and the self-aligning torque of the wheel, lateral acceleration, and vehicle lateral displacement are used to estimate the road friction coefficient during steering. Slalom and Double Line Change (DLC) tests in high friction conditions are conducted to verify the proposed estimation algorithm. The results show that the proposed method performs well during steering and the estimated road friction coefficient converges to the reference value rapidly.

In the study presented in [41] the virtual testing of intelligent driving is addressed by examining the key problems in modeling and simulating millimeter-wave radar environmental clutter, and proposing a modeling and simulation method for the environmental clutter of millimeter-wave radar in intelligent driving. Based on the attributes of millimeter-wave radar, the classification characteristics of the traffic environment of an intelligent vehicle and the generation mechanism of radar environmental clutter are analyzed. The statistical distribution characteristics of the clutter amplitude, the distribution characteristics of the power spectrum, and the electromagnetic dielectric characteristics are studied. Conditions such as road surface, rainfall, snowfall, and fog are deduced and designed. Experimental comparison results are utilized to validate the proposed model and simulation method.

## 9. Driver Assistance Systems and Automatic Vehicle Operation

**Figure 25:**
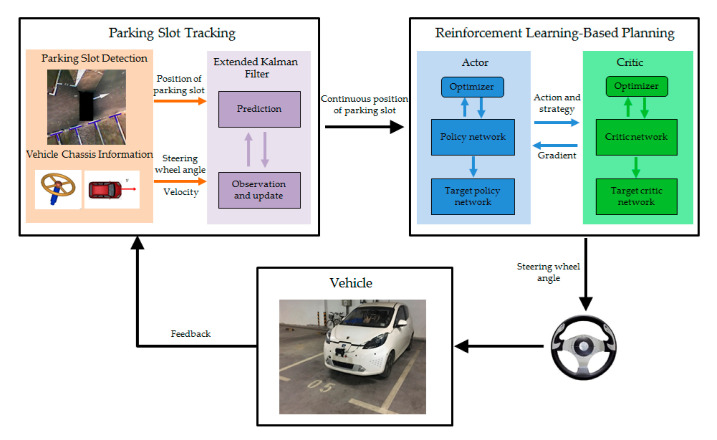
Overview of the reinforcement learning-based end-too-end parking method presented in [43].

The last three papers listed in the Special Issue are devoted to driver assistance systems and automatic vehicle operation. Thus, in [43] an automatic parking system (APS) based on reinforcement learning is presented. The parking path is planned based on the parking slot detected by the cameras. The path tracking module guides the vehicle to track the planned parking path. However, since the vehicle is a non-linear dynamic, path tracking error inevitably occurs, leading to the inclination and deviation of the parking. Accordingly, in the presented work, a reinforcement learning-based end-to-end parking algorithm is proposed to achieve automatic parking. The vehicle can continuously learn and accumulate experience from numerous parking attempts and then learn the command of the optimal steering wheel angle at different parking slots. Based on this end-to-end parking, errors caused by path tracking can be avoided. Moreover, to ensure that the parking slot can be obtained continuously in the process of learning, a parking slot tracking algorithm is proposed based on the combination of vision and vehicle chassis information (vehicle sensors). Furthermore, given that the learning network output is hard to converge, and it is easy to fall into local optimum during the parking process, several reinforcement learning training methods, in terms of parking conditions, are developed. Lastly, by the real vehicle test, it is proved that using the proposed method can achieve a better parking attitude than using the path planning and path tracking-based methods. An overview of the whole process is shown in Figure 25.

**Figure 26:**
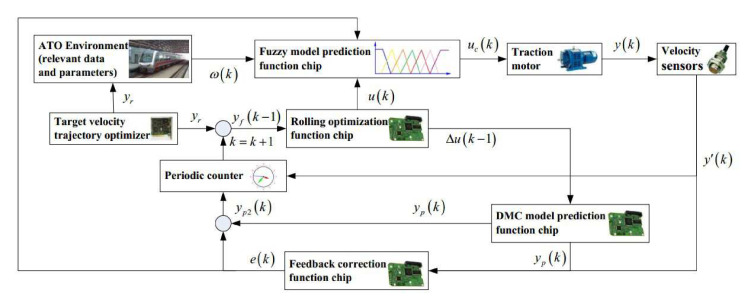
Schematic diagram of the Fuzzy Dynamic Matrix Control (DMC) Model Predictive Controller (MPC) proposed in [44] for automatic train operation.

Wang et al. present in [44] an improved model predictive controller based on the online obtaining of softness factor and fusion velocity for automatic train operation to enhance the tracking control performance. Specifically, the softness factor of the improved model predictive control algorithm is not a constant, conversely, an improved online adaptive adjusting method for softness factor based on fuzzy satisfaction of system output value and velocity distance trajectory characteristic is adopted, and an improved whale optimization algorithm has been proposed to solve the adjustable parameters (see Figure 26). The system output value for an automatic train operation is not sampled by a normal speed sensor. Instead, an online velocity sampled method for the system output value based on a fusion velocity model and an intelligent digital torque sensor is applied. The proposed strategies show a good performance in tracking precision, are simple and easily conducted, and can ensure the accomplishing of computational tasks in real-time. Finally, to verify the effectiveness of the model predictive controller, the MATLAB/Simulink and hardware-in-the-loop simulation (HILS) are adopted for automatic train operation tracking control, and the results also indicate that the proposed predictive controller has better tracking control effectiveness compared with the existing traditional model predictive controller.

In [42] the development of an intelligent driving assistant system based on vehicle telemetry and road accident risk map analysis is presented. The goal of the system is to alert the driver in order to avoid risky situations that may cause traffic accidents. In performance evaluations using real cars in real environments, the on-board intelligent assistant reproduced real-time audio-visual alerts. The method is mainly based on fuzzy reasoning and correctly warns the driver in real-time according to the telemetry data, the vehicle environment and the principles of safe driving practices and transportation regulation laws. Experimental results and conclusions emphasizing the advantages of the proposed intelligent driving assistant in the improvement of the driving task are obtained.

## 10. Conclusions

After analyzing all the manuscripts, some important and exciting conclusions arise. All the papers deal with well-known problems, which have been widely addressed by the research community during the last years. This is a clear example of the complexity of the problems involving sensors and sensing for intelligent vehicles. The issues most addressed in the Special Issue have been scene understanding, including object detection and tracking, and vehicle localization. We believe that it is a faithful representation of the importance of these problems for the advance of intelligent vehicles.

Fail-x systems (including fail-aware, fail-operational, fail-safe and fault diagnosis) are starting to emerge as an essential feature to be considered in future research works. Indeed, the ability to operate safely in the face of various types of failure and to be aware of and diagnose such failures, are fundamental issues in ensuring the safety of intelligent vehicles.

The complexity and variety of scenarios in which intelligent vehicles have to operate, as well as the need to function in the event of failure, are crucial elements that lead to most of the proposed solutions having a multi-sensor approach, including cameras, radar, LiDAR, IMUs, strains, vehicle sensors, and even smartphones as sensors onboard the vehicles. Intelligent vehicle sensing will be an “ever-present” problem, which will attract a lot of attention from both the research community and industry in the coming years. It is identified as the main bottleneck for enabling intelligent vehicles to operate autonomously and safely, and therefore to achieve good public acceptance.

On top of that, the current global situation, in which physical distance is socially necessary, is accelerating the need for intelligent and autonomous transportation, being the intelligent vehicles may be the most relevant topic in the field. We expect an increase in both the number of publications and the impact on this particular topic in the next coming years.
